# SELF-PERCEPTIONS OF AGING AS A PSYCHOLOGICAL RESOURCE FOR ENRICHMENT SEEKING

**DOI:** 10.1093/geroni/igad104.2710

**Published:** 2023-12-21

**Authors:** Faith-Christina Washington, Shannon Mejia, Wan Wen, Jessie Chin

**Affiliations:** University of Illinois, Urbana, Illinois, United States; University of Illinois Urbana-Champaign, Chanpaign, Illinois, United States; University of Illinois Urbana-Champaign, Urbana, Illinois, United States; University of Illinois at Urbana-Champaign, Champaign, Illinois, United States

## Abstract

In older adulthood, positive perceptions of one’s own aging can facilitate enrichment seeking–the underlying motivation to seek out new experiences and perform intellectually challenging activities–by diminishing and buffering reactions to perceived threats to one’s sense of self and perceived capabilities. In this study, we examined the extent to which self-perceptions of aging were associated with cognitive performance and affective experience while completing a series of challenging foraging tasks in the form of word puzzle games within the presence and absence of resources in the environment, such as a fireplace or an observing neurologist. The study employed a 2x2 within-person design (fireplace on vs. not; neurologist present vs. not) and took place within the home office of a home simulation environment. In each of the four conditions, 61 participants (age 50-82, 57% female) played the foraging task and then endorsed adjectives representing their feelings while playing that game. Multilevel models, which nested study stages within participants, showed individuals with higher self-perceptions of aging benefited significantly from both environmental manipulations, with the fireplace acting as environmental support and the observing neurologist raising the stakes of the task. However, despite providing additional motivation and being associated with stronger cognitive performance, the neurologist’s presence also provoked a higher negative affect response. The relationship between self-perceptions of aging and receptiveness to environmental resources merits further investigation to assess how certain environmental resources might act as a moderator of both cognition and affect.

